# Supplementary material for: Transplacental Therapeutic Drug Monitoring in Pregnant Women with Fetal Tachyarrhythmia Using HPLC-MS/MS
Source: Int J Mol Sci. 2023 Jan 17;24(3):1848. doi: 10.3390/ijms24031848 (PMC9916042; doi:10.3390/ijms24031848)

Table S1. The concentration of digoxin (ng/ml) in blood serum, urine and amniotic fluid at the observation points depending on the genotyping data of the pregnant woman according to ABCB1.

Groups of study, ng/ml	CC	Allele T	p-value
**1st point**			
Digoxin in blood*	1.5±0.5	1.7±0.6	0.583
Digoxin in urine**	459 (320;665)	579 (382,743)	0.354
Digoxin in amniotic fluid**	2.3 (1.9;2.6)	2.3 (1.3;3.5)	0.844
**2nd point**			
Digoxin in blood**	1.7 (1.2; 1.7)	1.8 (1.5; 2)	0.159
Digoxin in urine*	600±200	700±300	0.448
Digoxin in amniotic fluid*	2.1±0.8	4±2	0.127
**3rd point**			
Digoxin in blood*	1.9±0.3	1.8±0.5	0.681
Digoxine in urine*	900±200	600±400	0.12
**4th point (Delivery)**			
Digoxine in blood*	1.0±0.5	1.1±0.8	0.733
Digoxin in urine*	500±500	300±200	0.203
Digoxin in amniotic blood*	4.1±0.3	4±2	0.86
Digoxin in newborn blood*	0.4±0.2	0.5±0.4	0.265
*The data presented as M±SD (95% c.i.), variance analysis.<br>**The data presented as Me(Q1;Q3), Mann-Withey criteria			

Table S2. Intra- and interday precision and accuracy HPLC-MS/MS experiments (three different days) at four digoxin/sotalol concentrations (LOQ, low QC, medium QC, and high QC) in plasma, urine and amniotic fluid.

**Analyte**	**Matrix**	**QC, type**	**QC, concentration, ng/ml (digoxin) and μg/ml (sotalol)**	**Intraday precision (RSD, %, n=5)**			**Intraday accuracy (%, n=5)**			**Interday precision (RSD, %,1)**	**Interday accuracy (%, n=3)**
				**1 day**	**2 day**	**3 day**	**1 day**	**2 day**	**3 day**		
Digoxin	Plasma	LOQ	0.6	3.76	2.24	1.80	-8.22	-11.15	-12.90	3.10	-10.80
		low QC	1.8	7.94	6.47	4.25	5.32	-3.38	-11.43	9.10	-3.20
		medium QC	5.0	1.00	2.89	6.30	-14.06	-10.72	-9.07	4.20	-11.30

		high QC	7.5	7.91	1.93	9.75	1.65	-12.90	1.01	9.60	-3.40
		LOQ	0.2	2.98	1.07	14.40	15.33	18.43	0.62	12.00	11.50
		low QC	0.6	9.30	5.38	2.93	3.81	6.39	-12.41	10.40	-0.70
		medium QC	5.0	10.65	2.20	3.03	6.69	11.52	-11.40	12.10	2.30
Sotalol	Plasma	high QC	7.5	5.90	5.68	10.12	5.74	4.35	-6.14	8.90	1.30
		LOQ	10.0	0.24	1.72	1.34	-14.12	12.74	11.89	12.60	4.50
		low QC	25.0	10.72	12.16	8.71	7.42	1.62	-6.32	10.90	0.00
		medium QC	200.0	6.69	8.52	8.11	4.59	-2.70	-8.45	7.90	-3.10
Digoxin	Urine	high QC	500.0	10.25	6.78	6.51	6.69	-4.52	-6.87	9.60	-1.60
		LOQ	10.0	2.03	5.55	7.66	-13.20	-5.52	-2.71	6.70	-7.10
		low QC	30.0	1.38	2.13	8.40	13.41	-13.21	-4.71	12.30	-1.50
		medium QC	500.0	1.24	5.39	7.25	12.52	-0.68	6.89	7.70	6.20
Sotalol	Urine	high QC	750.0	3.21	2.22	9.47	12.09	10.46	-1.59	8.70	7.00
		LOQ	0.5	4.63	4.99	2.15	5.15	-5.44	-12.70	8.20	-4.30
		low QC	1.5	7.67	7.05	11.66	-7.05	-6.37	1.52	9.20	-4.00
		medium QC	5.0	4.68	6.89	10.60	-9.16	-7.67	0.39	8.30	-5.50
Digoxin	Amniotic fluid	high QC	7.5	2.46	4.50	8.34	-12.81	-9.18	1.55	7.90	-6.80

Sotalol	Amniotic fluid	LOQ	0.2	13.33	11.21	8.35	-6.65	-0.68	7.10	11.90	-0.10
		low QC	0.6	5.67	10.14	6.92	10.59	6.71	5.18	7.70	7.50
		medium QC	5.0	7.77	4.17	11.21	7.75	6.47	-4.33	9.60	3.30
		high QC	7.5	5.40	3.77	4.85	10.29	6.81	6.54	4.80	7.90

Table S3. Matrix effect and extraction recovery results for three QC levels (LOQ, medium QC, and high QC).

**Analyte**	**Matrix**	**Matrix effect (ME, n=3), %**			**Recovery (ME, n=3), %**		
		**LLOQ**	**Medium QC**	**High QC**	**LLOQ**	**Medium QC**	**High QC**
Digoxin	Plasma	76	110	95	75	96	78
	Urine	85	92	89	80	97	85
Sotalol	Plasma	81	114	88	77	98	85
	Urine	88	83	94	81	83	101

Table S4. The results of HPLC-MS/MS MRM methods validation.

Analyte	Digoxin			Sotalol		
Matrix	Plasma	Amniotic fluid	Urine	Plasma	Amniotic fluid	Urine
Calibration curve, equation	y=0.39034*x -0.989	y=0.18864*x +0.01461	y=0.01519*x +0.00919	y=0.29340*x +0.02059	y=0.19969*x +0.00537	y=0.01417*x-0.00754
Calibration curve, r	0.99605	0.99868	0.99537	0.99419	0.99397	0.99325
LLOD, ng/ml (digoxin) and µg/ml (sotalol)	0.2	0.2	7.5	0.2	0.2	7.7
LLOQ, ng/ml (digoxin) and µg/ml (sotalol)	0.2	0.5	7.5	0.2	0.2	10.0
Selectivity (STD of the peak area, %)	14.5	10.5	10.5	12.5	9.6	9.6
Matrix effect	93.7±13.9	95.0±4.9	95.0±4.9	91.3±10.1	98.0±11.4	98.0±11.4

(ME±STD), %						
Recovery (ME±STD), %	82±7.2	89.7±5.2	89.7±5.2	86.7±8.7	88.7±8.7	88.7±8.7
Accuracy, %	(-11.5; -3.2)	(-6.8; - 4)	(-3.1; 4.5)	(-0.7; 11.5)	(-0.1; 7.9)	(-7.1, 7)
Precision, %	(3.1; 9.6)	(7.9; 9.2)	(7.9; 12.6)	(8.9; 12.1)	(4.8; 11.9)	(6.7; 12.3)
Robustness (STD of the peak area, %)	4.0	1.5	1.5	3.5	2.0	2.0

FigureS1: Calibration curve for digoxin in amniotic fluid. Healthy women urine was used as a matrix.

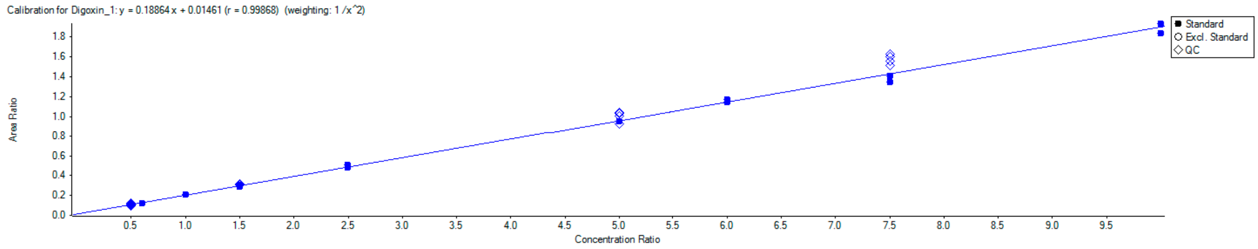

FigureS2: Chromatograms for blank and samples in amniotic fluid for digoxin. The healthy women urine was used as a blank.

1 – Samples:

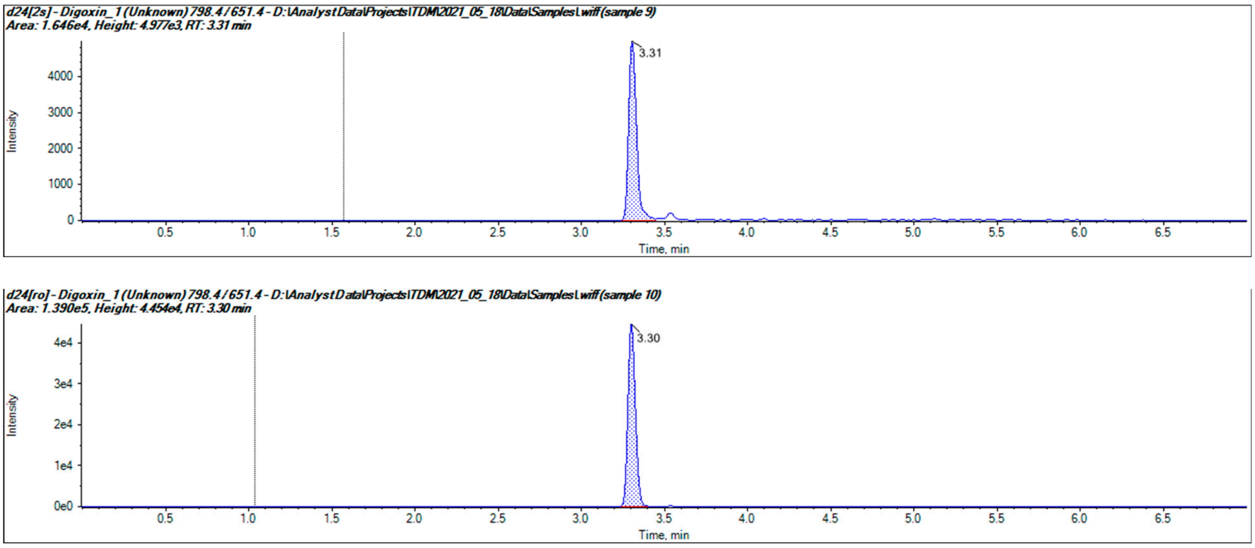

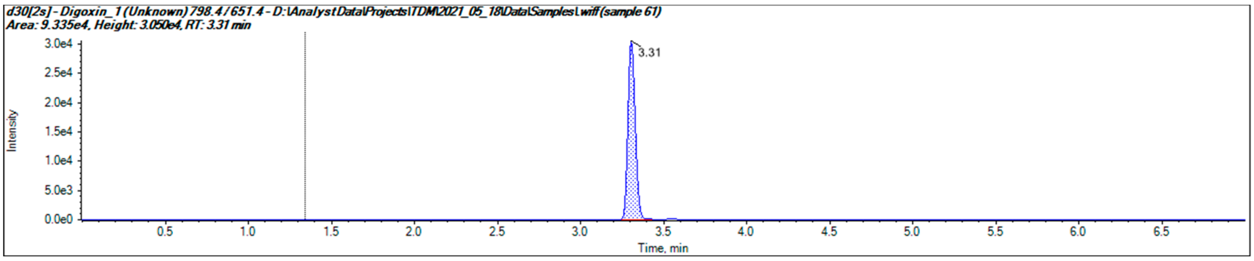

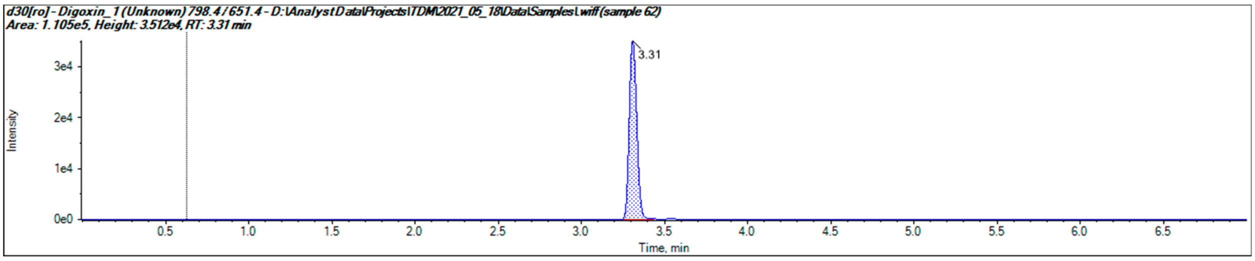

2 – Blank:

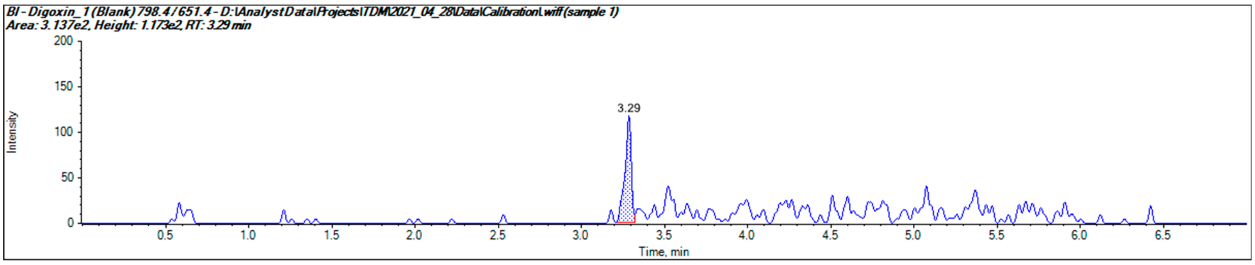

3 – Calibration levels and QC:

$Cal_{1Digoxin}$=0.5 ng/ml

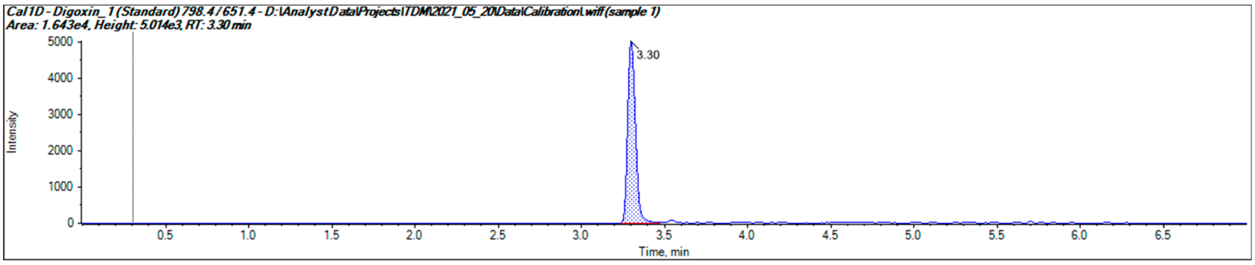

$Cal_{7Digoxin}$=10 ng/ml

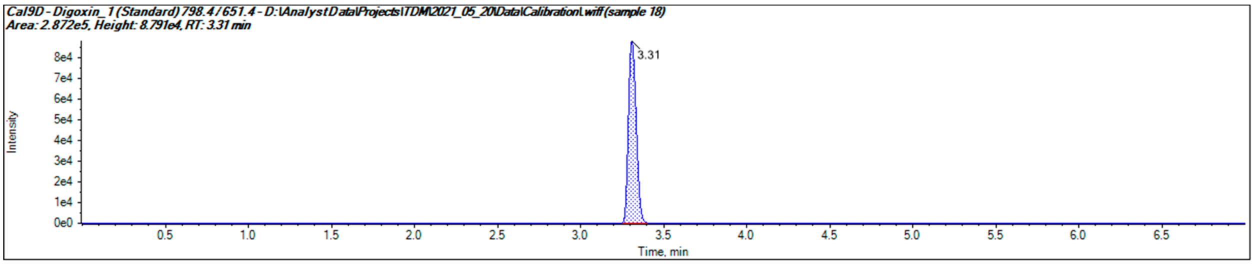

$QC_{1Digoxin}$=0.6 ng/ml

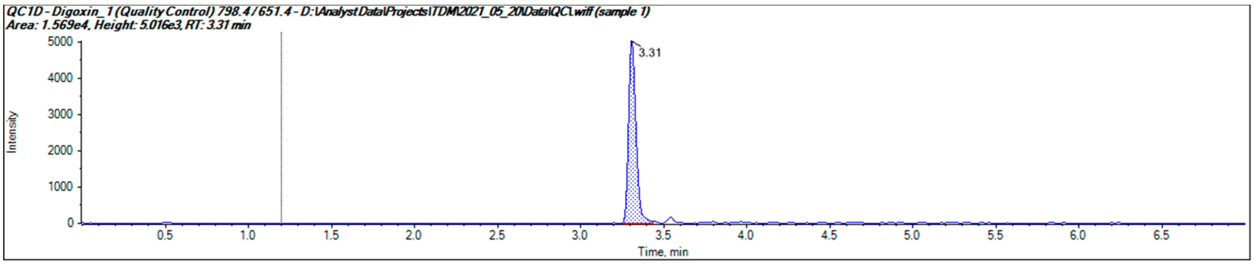

QC$_{4Digoxin}$=7.5 ng/ml

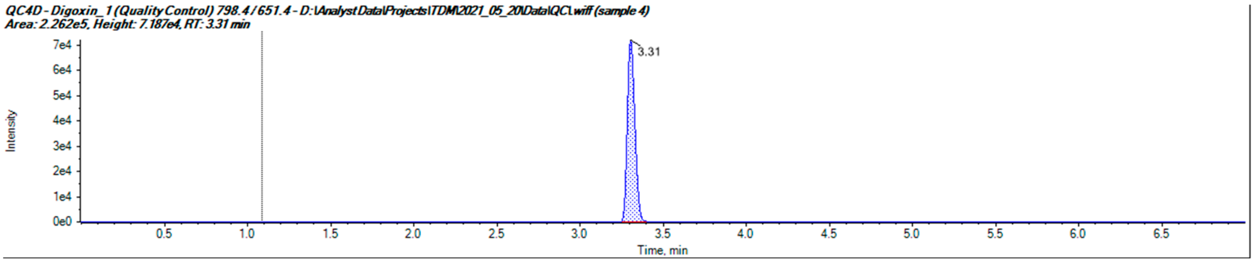

FigureS3: Calibration curve for digoxin in blood. The healthy women plasma was used as a matrix.

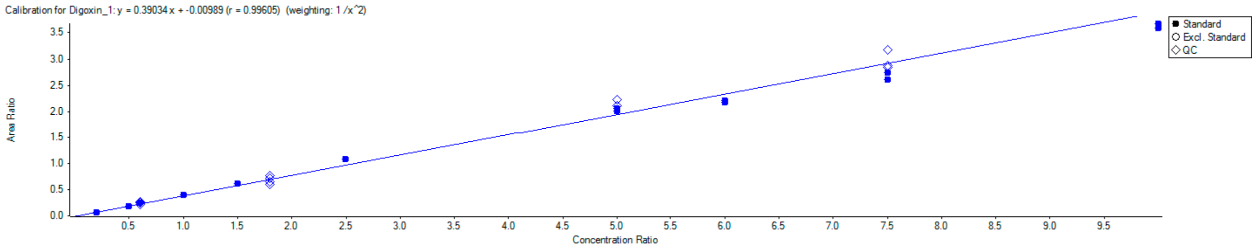

FigureS4: Chromatograms of blank and samples for digoxin on blood.

1 – Samples:

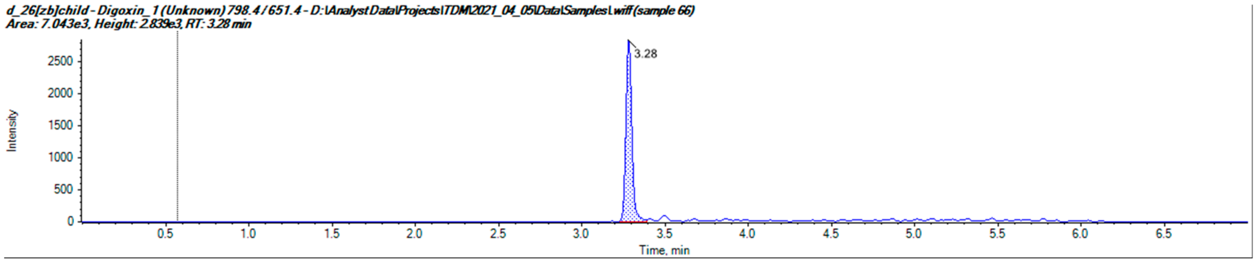

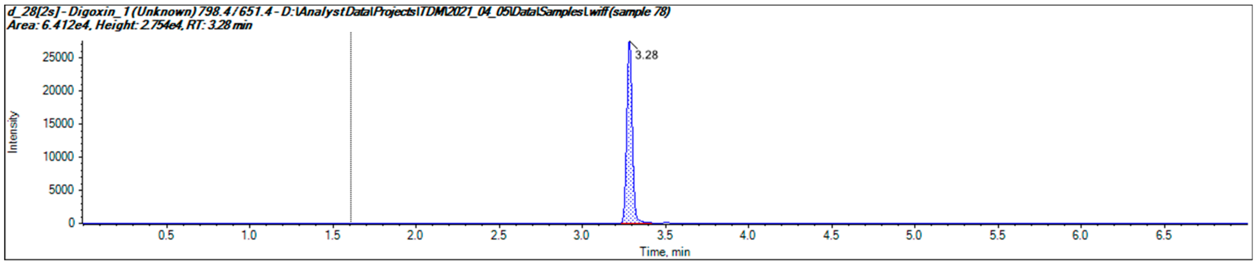

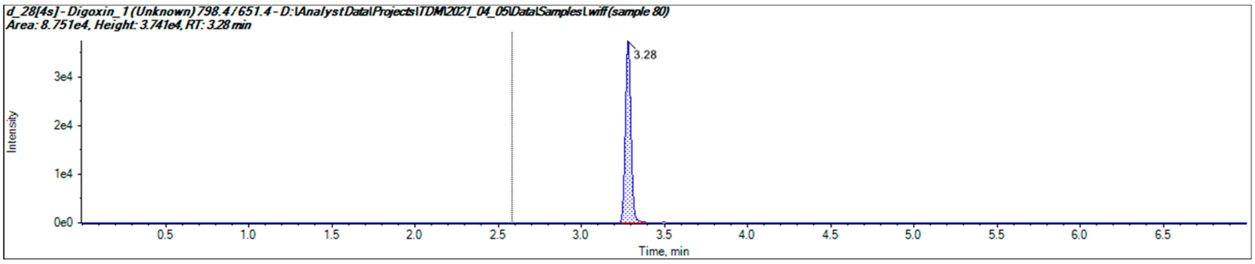

2 – Blank:

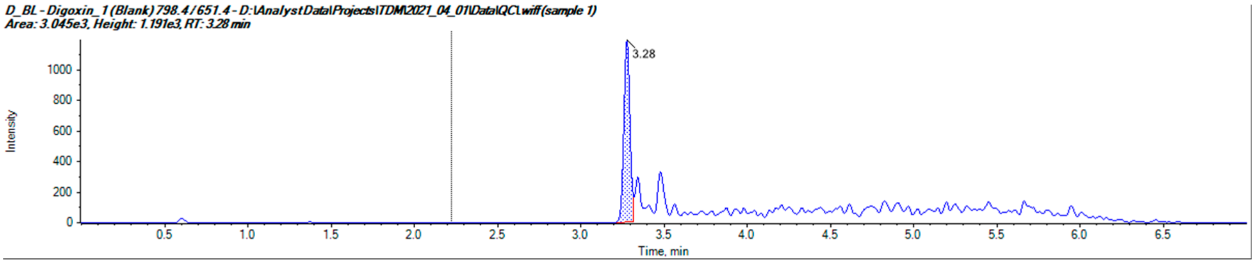

3 – Calibration levels and QC:

$Cal_{1Digoxin}$=0.2 ng/ml

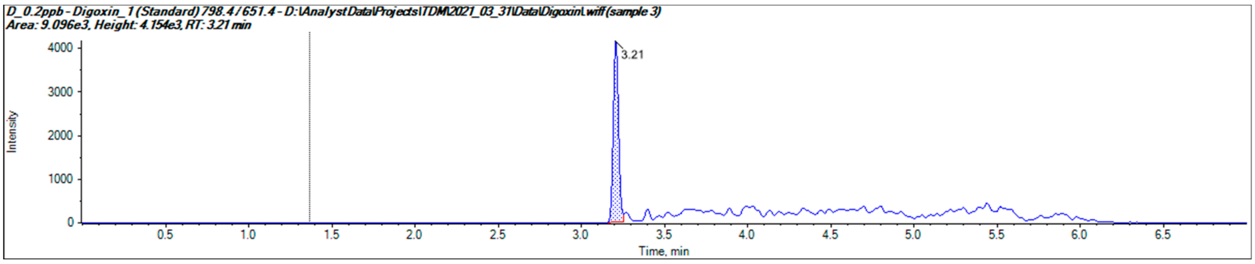

$Cal_{2Digoxin}$=0.5 ng/ml

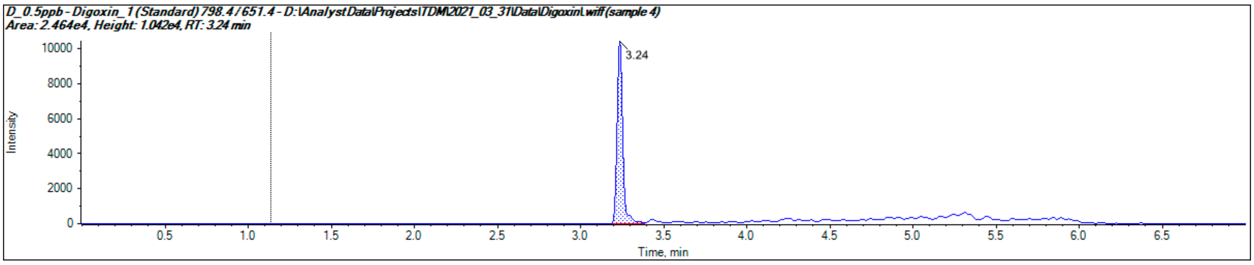

$Cal_{3Digoxin}$=0.6 ng/ml

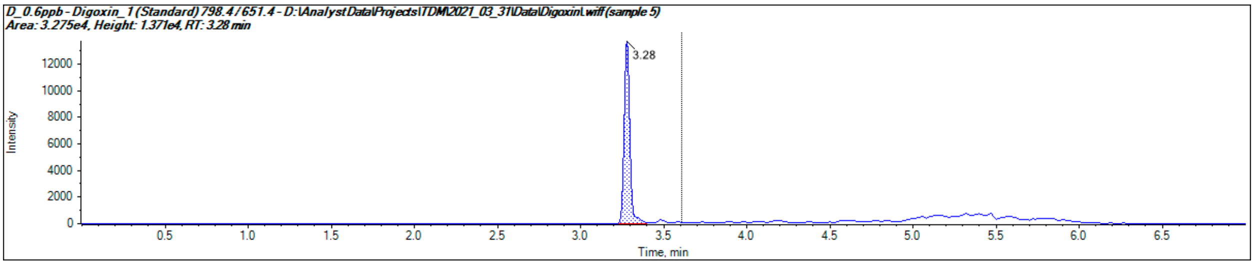

$Cal_{1Digoxin}$=10 ng/ml

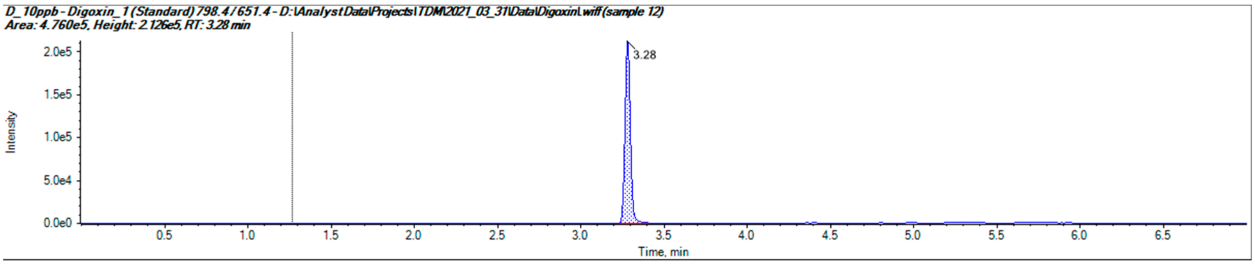

QC$_{1Digoxin}$=0.6 ng/ml

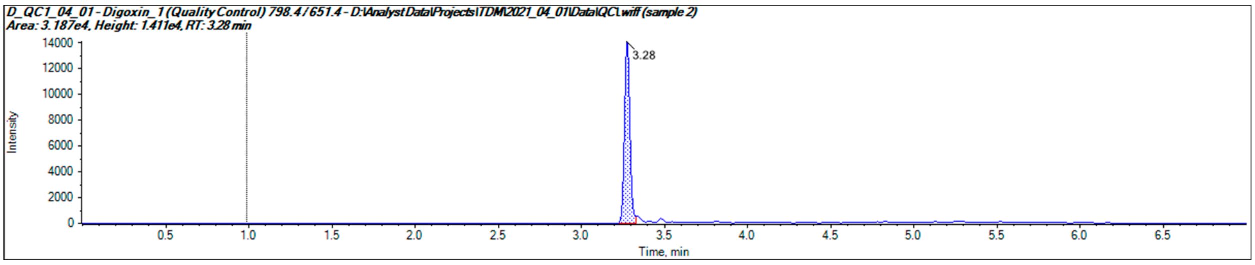

QC$_{2Digoxin}$=1.8 ng/ml

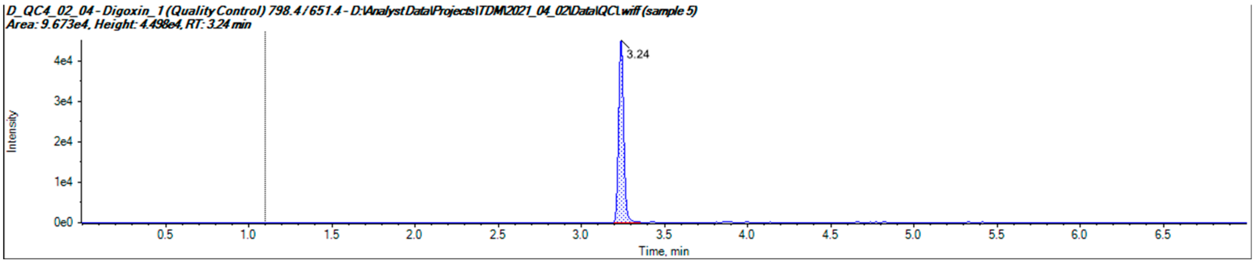

QC$_{4Digoxin}$=7.5 ng/ml

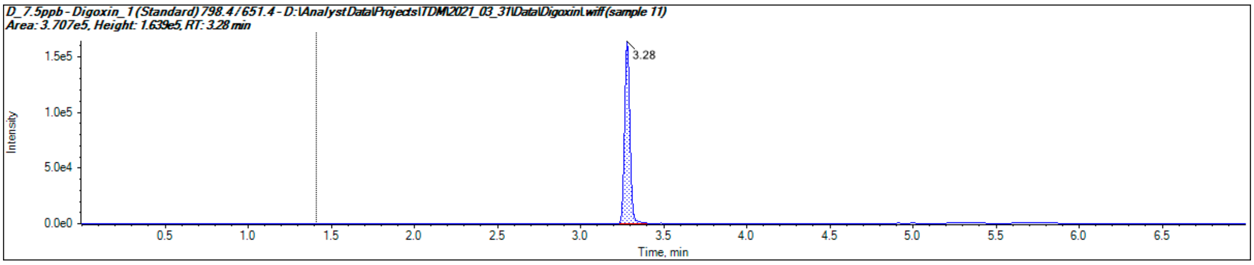

FigureS5: Calibration curve for digoxin in urine. The healthy women urine was used as a matrix.

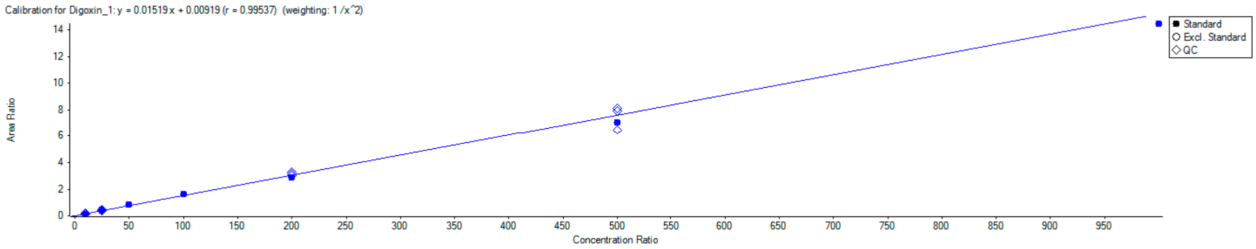

FigureS6: Chromatograms for blank and samples. The healthy women urine was used as a blank.

1 – Samples:

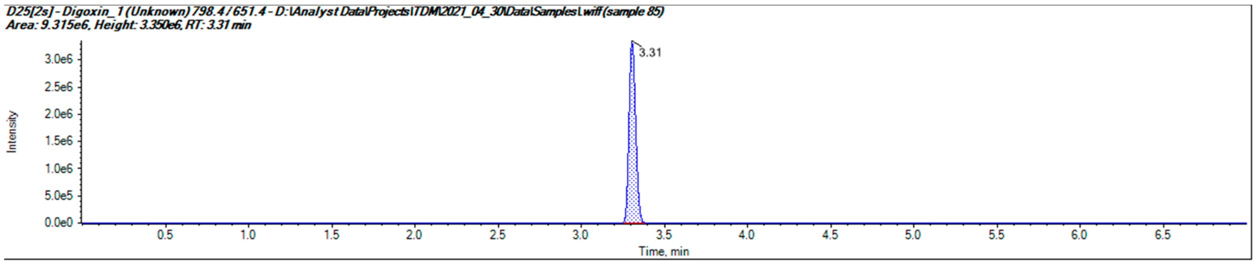

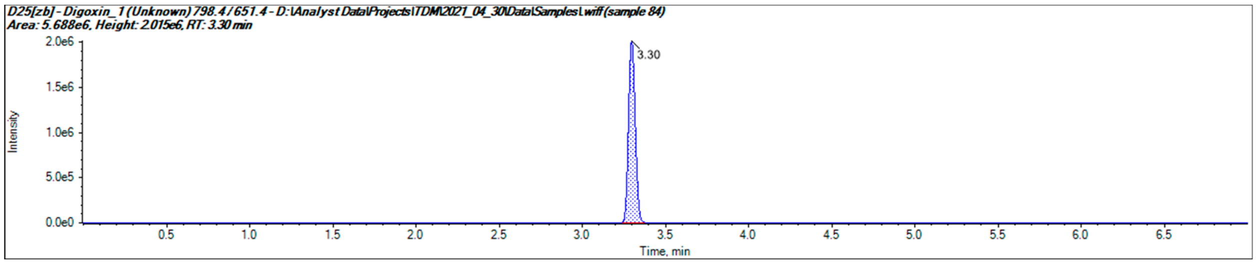

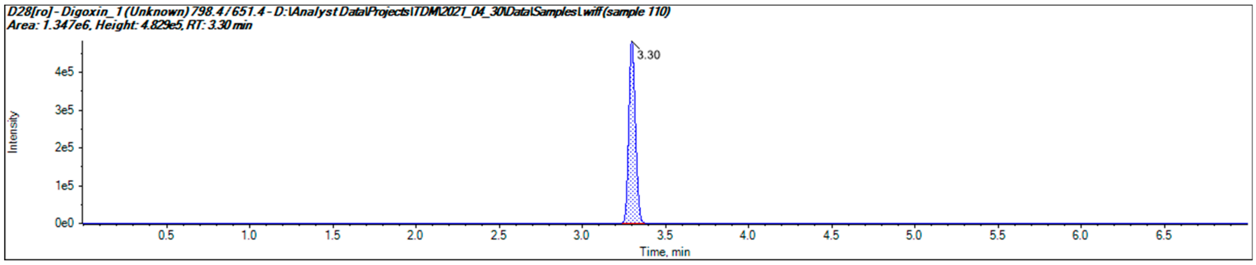

2 – Blank:

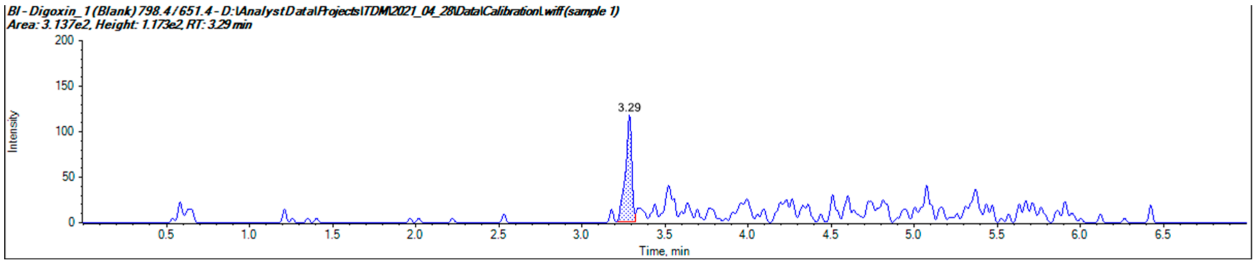

3 – Calibration levels:

Cal1D - Digoxin_1 (Standard) 798.4 / 651.4 - D:\Analyst Data\Projects\TDM\2021_05_06\Data\Calibration\.wiff (sample 2)
Area: 1.309e5, Height: 4.506e4, RT: 3.30 min

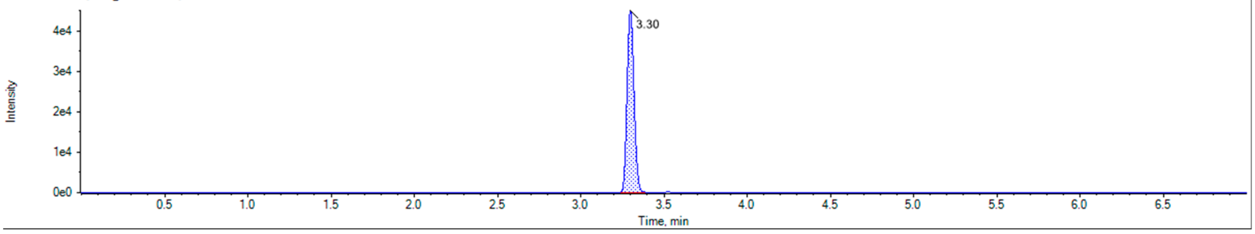

Cal7D - Digoxin_1 (Standard) 798.4 / 651.4 - D:\Analyst Data\Projects\TDM\2021_05_06\Data\Calibration\.wiff (sample 9)
Area: 1.187e7, Height: 3.854e6, RT: 3.30 min

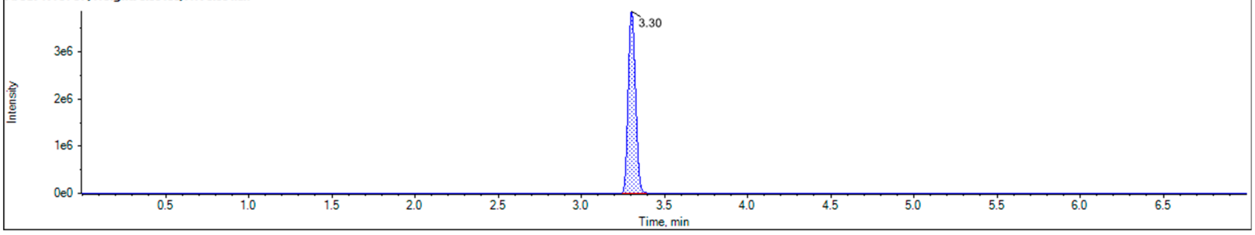

FigureS7: Calibration curve for sotalol in amniotic fluid. The healthy women urine was used as a matrix.

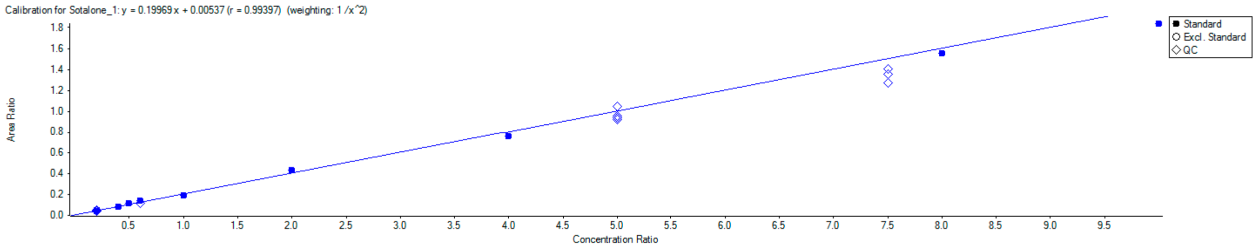

FigureS8: Chromatograms for blank and samples in amniotic fluid for sotalol. The healthy women urine was used as a blank.

1 – Samples:

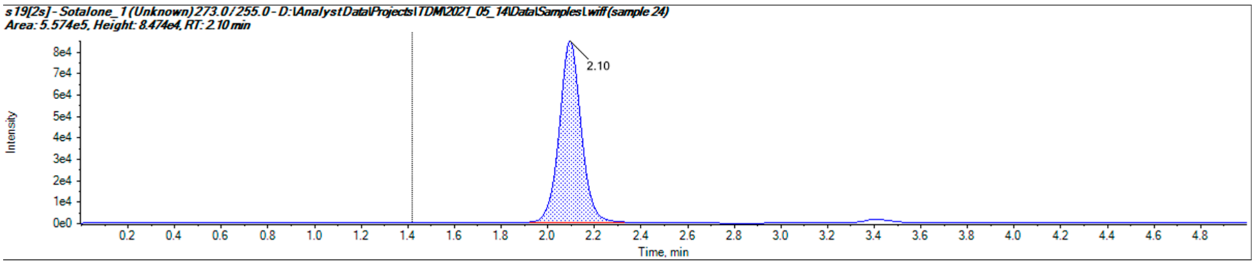

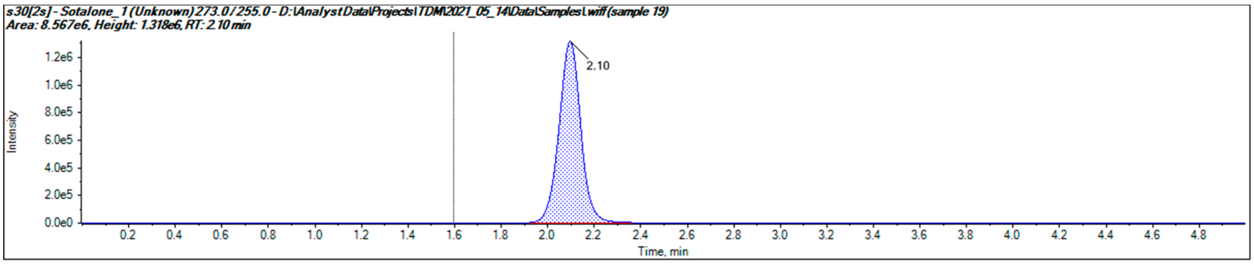

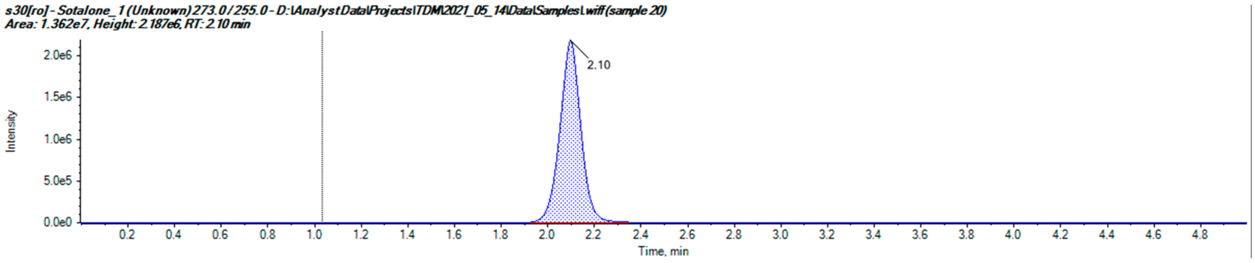

2 – Blank:

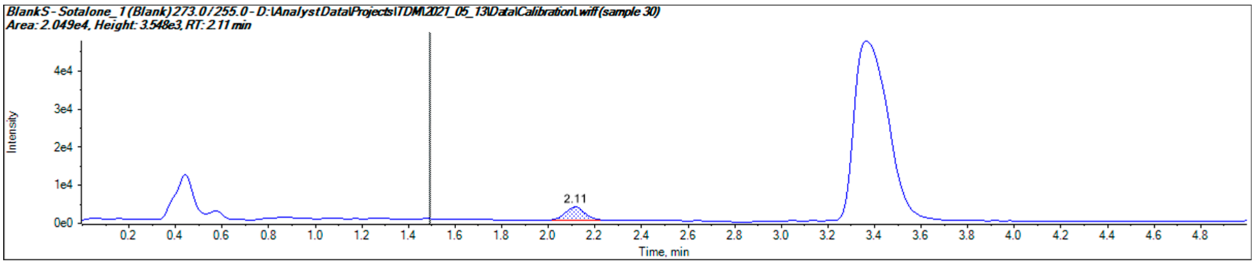

3 – Calibration levels and QC:

Cal$_{1\text{Sotalol}}$=0.2 µg/ml

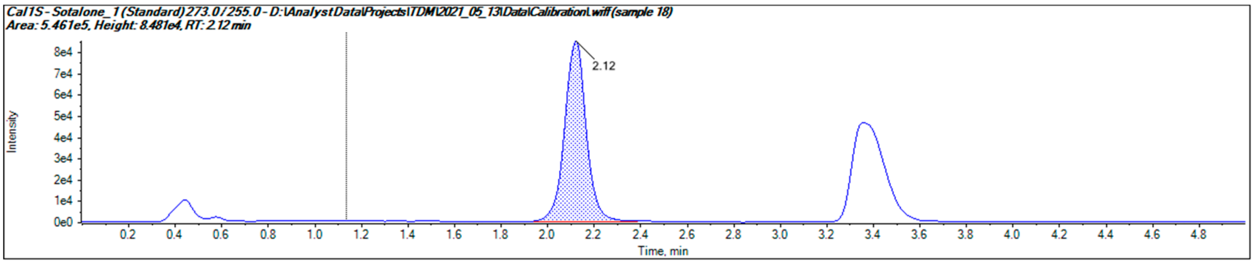

Cal$_{9\text{ Sotalol}}$ =10 µg/ml

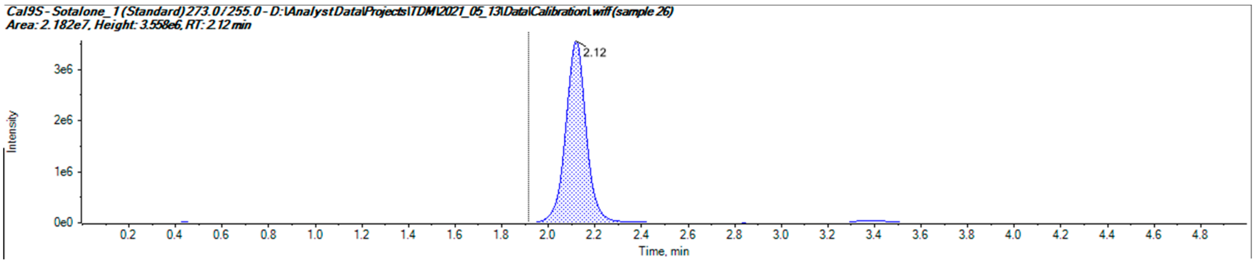

QC$_{1\text{Sotalol}}$=0.2 µg/ml

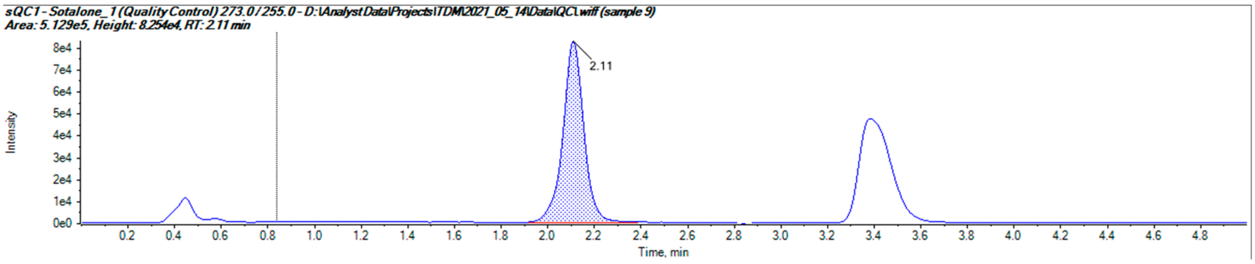

QC$_{4\text{Sotalol}}$=7.5 µg/ml

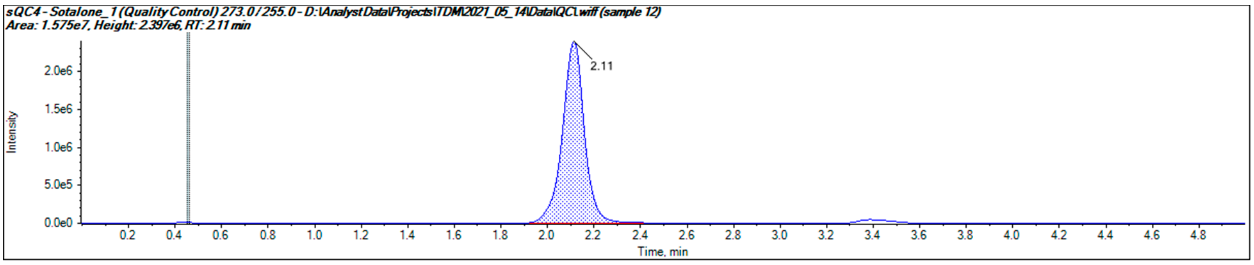

FigureS9: Calibration curve for sotalol in blood. The healthy women plasma was used as a matrix.

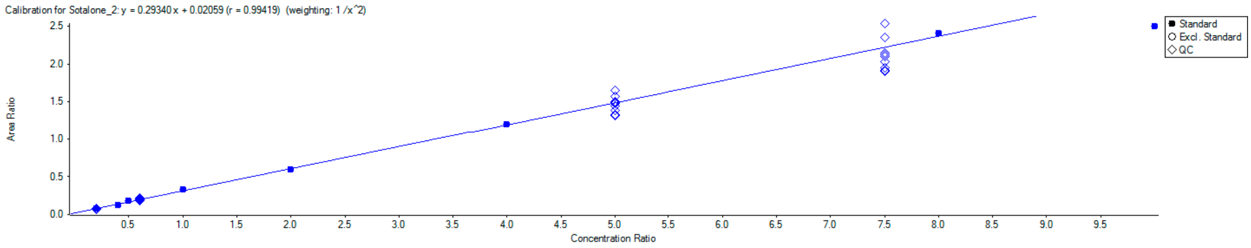

FigureS10: Chromatograms for blank and samples. The healthy women plasma was used as a blank.

1 – Samples:

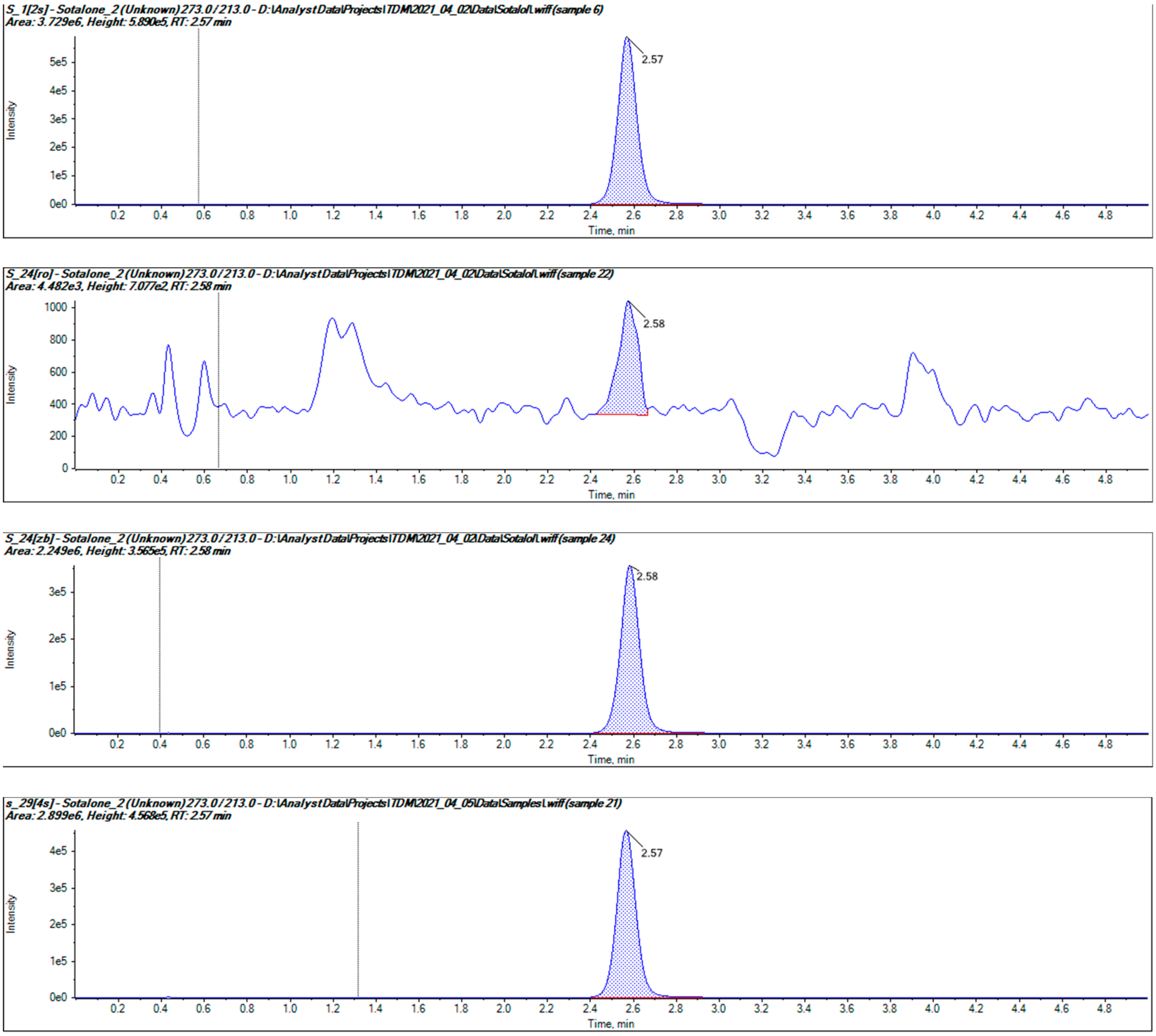

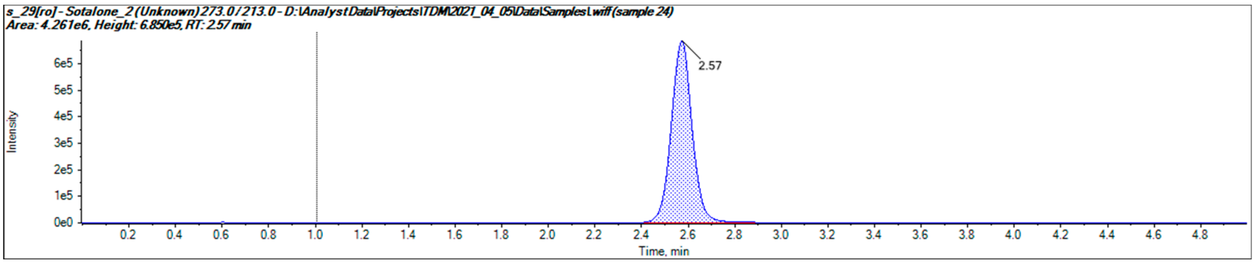

2 – Blank:

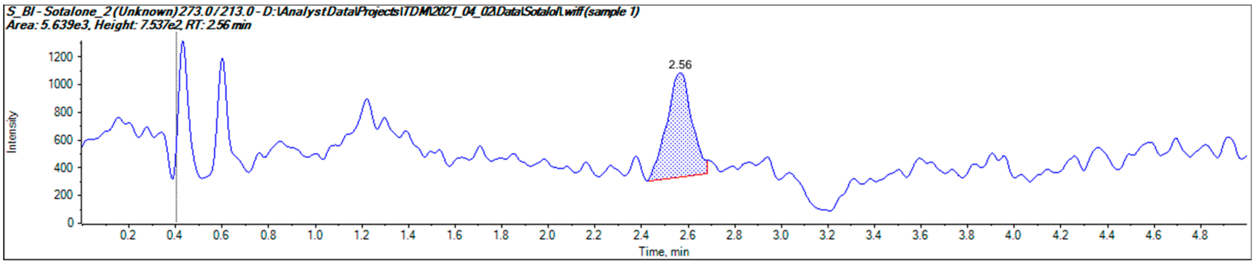

3 – Calibration levels:

Cal1Sotalol=0.2 µg/ml

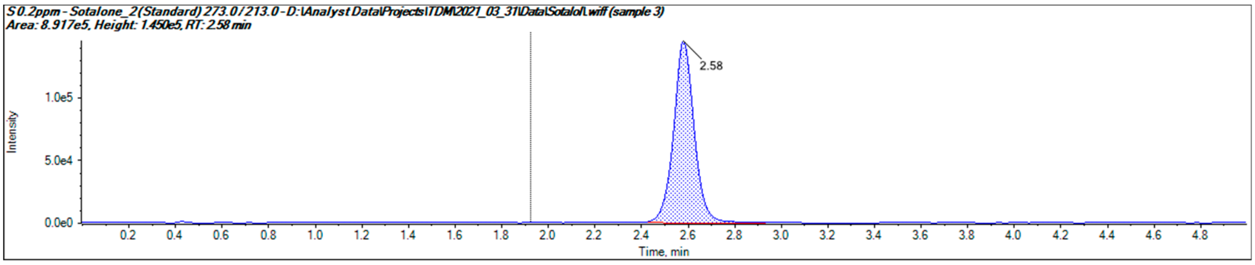

Cal9Sotalol=10 µg/ml

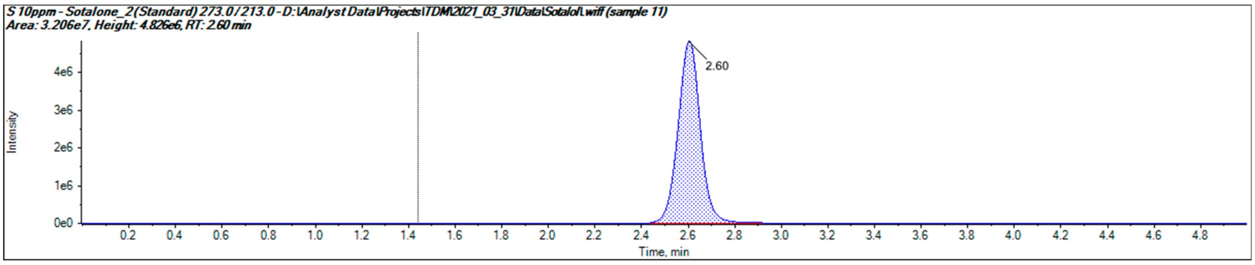

QC1Sotalol=0.2 µg

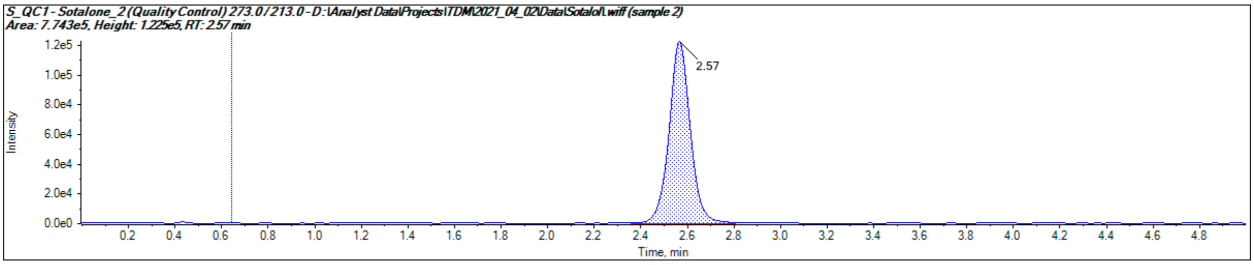

QC4Sotalol=7.5 µg/ml

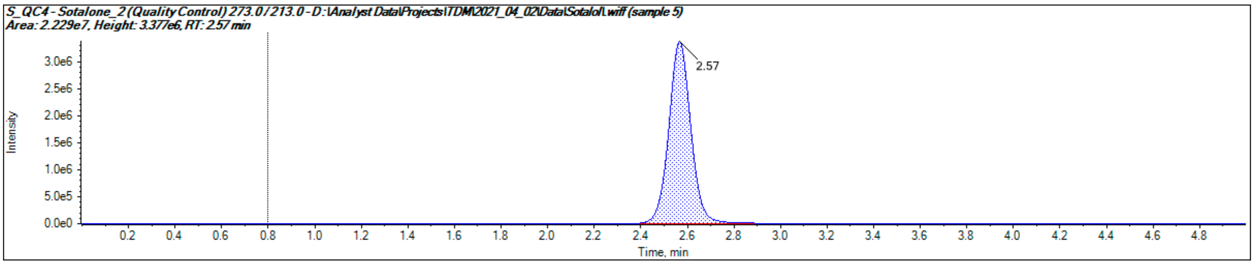
S_QC4 - Sotalone_2 (Quality Control) 273.0 / 213.0 - D:\Analyst Data\Projects\TDM\2021_04_02\Data\Sotalol\.wiff (sample 5)
Area: 2.229e7, Height: 3.377e6, RT: 2.57 min
Intensity
Time, min
2.57

FigureS11: Calibration curve for sotalol in urine. The healthy women urine was used as a matrix.

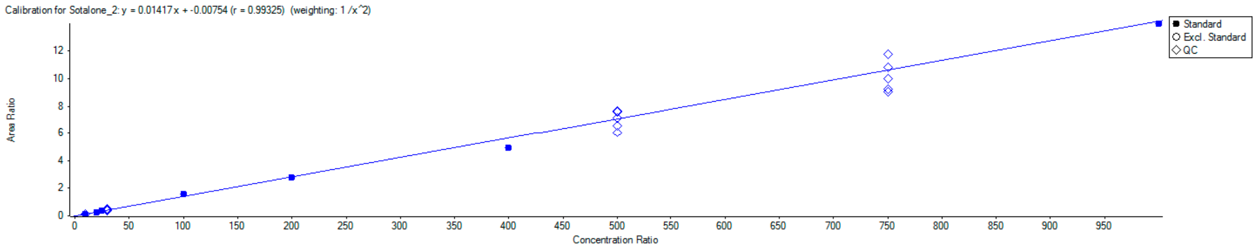

FigureS12: Chromatograms for blank and samples. The healthy women urine was used as a blank.

1 – Samples:

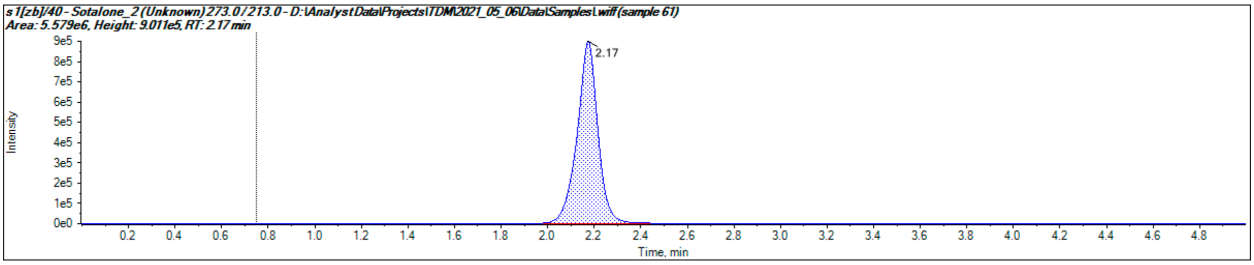

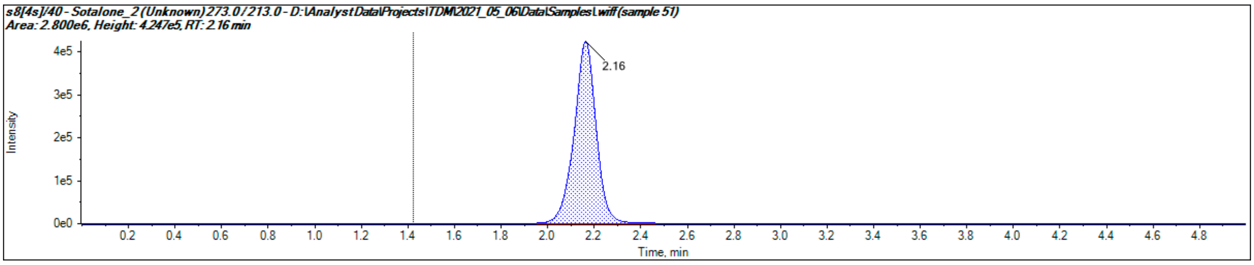

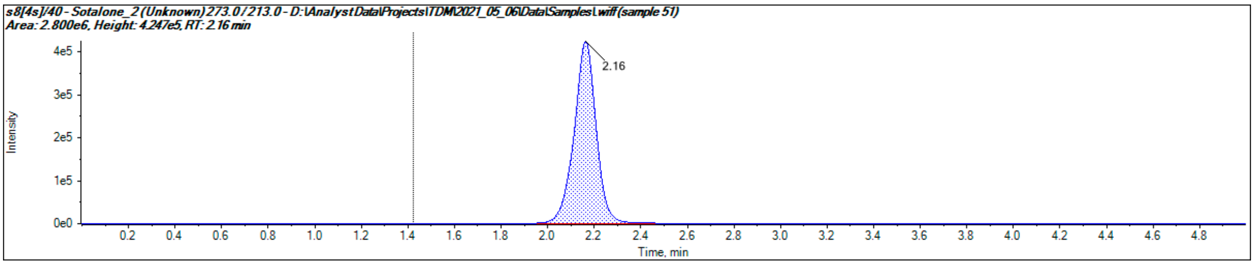

2 – Blank:

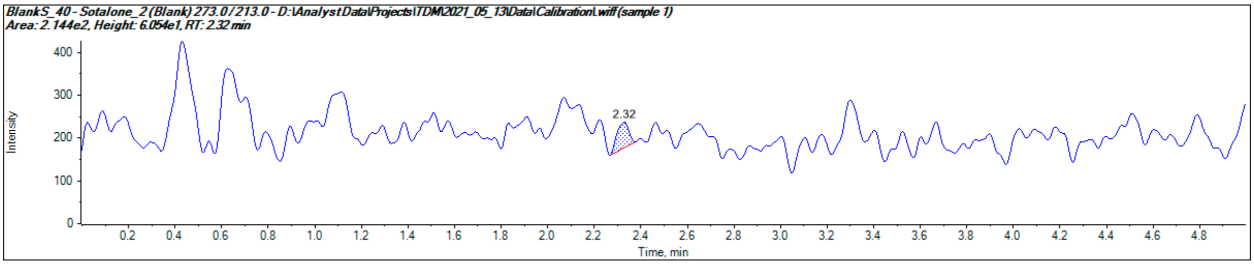

3 – Calibration levels:

$Cal_{1Sotalol}$=10 µg/ml

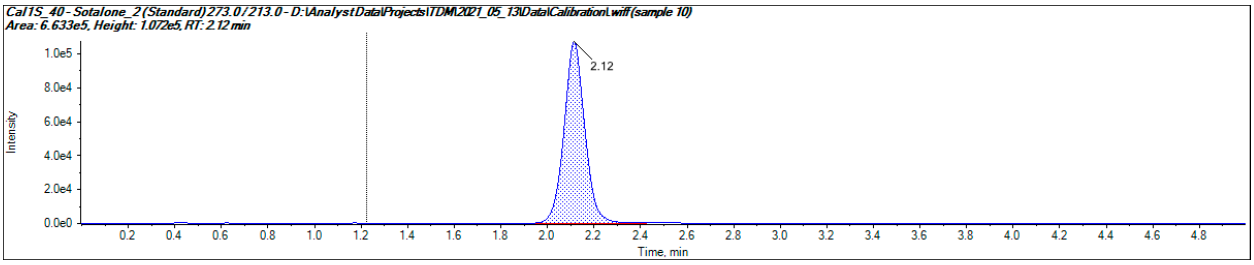

Cal$_{7Sotalol}$=1000 µg/ml

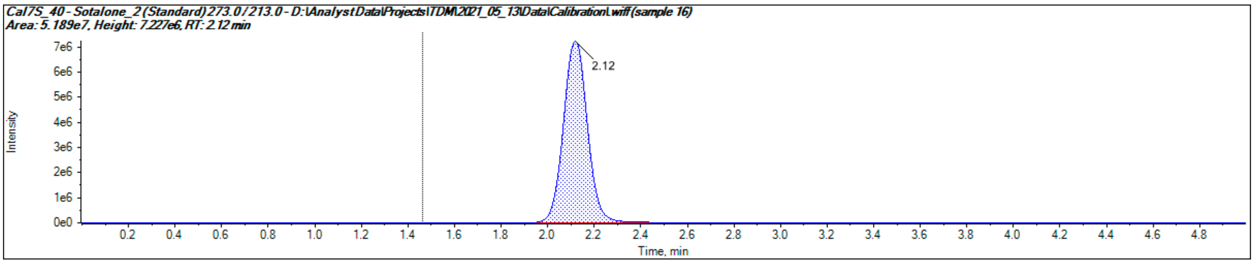

Supplement: Supplementary file 1 [file ijms-24-01848-s001.zip › Supplementary File S1.pdf]
